# Editorial on special issue: “Metamaterials and plasmonics in Asia”

**DOI:** 10.1515/nanoph-2022-0226

**Published:** 2022-04-26

**Authors:** Tie Jun Cui, Jeong Weon Wu, Teruya Ishihara, Lei Zhou

**Affiliations:** Southeast University, Nanjing, China; Ewha Womans University, Seoul, Korea; Tohoku University, Sendai, Japan; Fudan University, Shanghai, China

The special issue “Metamaterials and Plasmonics in Asia” was developed from the fifth A3 Metamaterials Forum held in Nanjing, China, on 26–29 June 2021. The A3 Metamaterials (A3 META) Forum is an annual meeting for leading researchers working on metamaterials in three Asian countries (Korea, Japan and China). Topics of the forum include artificial materials and surfaces in electromagnetic, acoustic and other systems.

The first A3 Metamaterials Forum was launched in 2016 in Sendai, Japan, led by Prof. Jeong Weon Wu, Prof. Teruya Ishihara and Prof. Lei Zhou. The forum was then successfully held in Shanghai, China, in 2017; Pohang, Korea, in 2018; and Sapporo, Japan, in 2019. The fifth A3 Metamaterials Forum was originally scheduled to be held in Nanjing, China, in 2020, but was postponed to 2021 and held in a hybrid format due to the COVID-19 pandemic. Speakers from Japan, Korea and Hong Kong SAR, China, were invited to speak online.

Ten review articles in this special issue are presented by leading researchers on a variety of topics. Qin et al. overviewed the underlying physics of waveguide effective plasmonics in lower frequencies based on the structural dispersion, and their applications in novel effective plasmonic devices [1]. Otsuji et al. focused on the graphene-based plasmonic metamaterials for terahertz laser transistors [2]. Tian et al. introduced the recent advances in microwave metamaterials for simultaneous wireless information and power transmissions [3]. Deng et al. reviewed multi-freedom optical metasurfaces and their applications in vectorial holography [4]. Koala et al. overviewed the nanophotonics-inspired all-silicon waveguide platforms for terahertz integrated systems [5]. Du et al. introduced the latest research progress in multifunctional and tunable optical metasurfaces [6]. Lee et al. discussed the perspectives of broadband metasurfaces and the applications in photo-electric tweezers [7]. Park et al. reviewed the free-form optimisation of nanophotonic devices via classical methods and deep learning [8]. Ma et al. presented a comprehensive overview of the optical generation for strong-field terahertz radiation and its applications in nonlinear terahertz metasurfaces [9]. Han et al. discussed the recent progress in responsive photonic nanopixels with metallic and dielectric nanoscatters [10].

The issue also presents 31 original research papers. The research papers can be roughly categorised as follows, although there are certain inevitable overlaps between different categories:

1. Optical surface plasmons [11], [12], [13]
2. Spoof surface plasmons at microwave frequencies [14]
3. Optical and infrared metasurfaces [15], [16], [17], [18], [19], [20]
4. Microwave metasurfaces [21], [22], [23], [24], [25]
5. Terahertz metasurfaces and technologies [26], [27], [28], [29], [30], [31]
6. Right/left-handed metamaterial lines [32]
7. Optical theories and technologies [33], [34], [35], [36], [37], [38]
8. Topological photonics [39, 40]
9. Acoustic metamaterials [41]

Metamaterial research has been in the spotlight for several decades since the concept of negative refraction was firstly proposed by Veselago in 1968 [42] and fundamental theoretical and experimental research was performed by Sir John Pendry and David Smith in the late 1990s and early 2000s [43, 44]. Recently, metasurfaces [45, 46], plasmonic metamaterials [47, 48] and information metamaterials [49], [50], [51] have become hot topics and exhibited great application potential. From this special issue, we are glad to see endeavours ranging from optics to microwaves and electromagnetics to acoustics. There are some scientific breakthroughs in electromagnetic vortices and topological photonics that bring new ideas and possibilities to the field. Meanwhile, more engineering attempts emerge for applications, especially in the microwave community. Besides elaborately designed and applicable devices, some systematic prototypes have also been developed for wireless communications and sensing.

We sincerely appreciate all contributions from the authors to this special issue and believe that it provides an overview of recent efforts by leading scientists in the field of metamaterials in Asia.
